# Collecting specialty-related medical terms: Development and evaluation of a resource for Spanish

**DOI:** 10.1186/s12911-021-01495-w

**Published:** 2021-05-04

**Authors:** Pilar López-Úbeda, Alexandra Pomares-Quimbaya, Manuel Carlos Díaz-Galiano, Stefan Schulz

**Affiliations:** 1 Universidad de Jaén, Campus Las Lagunillas, s/n, 23071 Jaén, Spain; 2 Medical University of Graz, Auenbruggerpl No 2, 8036 Graz, Austria; 3 Pontificia Universidad Javeriana, Cra. 7 No 40-62, Bogotá, 110231 Colombia

**Keywords:** Natural language processing, Vocabulary, Medical sub-language, Clinical specialty, Medical sub-domain

## Abstract

**Background:**

Controlled vocabularies are fundamental resources for information extraction from clinical texts using natural language processing (NLP). Standard language resources available in the healthcare domain such as the UMLS metathesaurus or SNOMED CT are widely used for this purpose, but with limitations such as lexical ambiguity of clinical terms. However, most of them are unambiguous within text limited to a given clinical specialty. This is one rationale besides others to classify clinical text by the clinical specialty to which they belong.

**Results:**

This paper addresses this limitation by proposing and applying a method that automatically extracts Spanish medical terms classified and weighted per sub-domain, using Spanish MEDLINE titles and abstracts as input. The hypothesis is biomedical NLP tasks benefit from collections of domain terms that are specific to clinical subdomains. We use PubMed queries that generate sub-domain specific corpora from Spanish titles and abstracts, from which token n-grams are collected and metrics of relevance, discriminatory power, and broadness per sub-domain are computed. The generated term set, called Spanish core vocabulary about clinical specialties (SCOVACLIS), was made available to the scientific community and used in a text classification problem obtaining improvements of 6 percentage points in the F-measure compared to the baseline using Multilayer Perceptron, thus demonstrating the hypothesis that a specialized term set improves NLP tasks.

**Conclusion:**

The creation and validation of SCOVACLIS support the hypothesis that specific term sets reduce the level of ambiguity when compared to a specialty-independent and broad-scope vocabulary.

## Background and contributions

### Limitations of language resources for the analysis of clinical narratives

Information extracted from clinical narratives has been used for a wide range of biomedical applications [1–4], and natural language processing (NLP) and machine learning (ML) techniques have evolved as important parts of clinical information extraction initiatives [5]. Information extraction supports a wide variety of clinical and research use cases, such as building disease-specific cohorts [5], processing and analyzing mentions of signs and symptoms [6, 7], detecting and assessing adverse drug events and risks [8–10], extracting key information for reporting or quality assurance [11–14], among others.

Most of these studies rely on controlled vocabularies (CVs) in different flavors, known as dictionaries, lexicons, terminologies and ontologies[15]. They are curated by experts and public bodies and are mostly tailored to specific purposes like disease or adverse event reporting, annotation of health records for billing, data collection for clinical research, and literature indexing. They exhibit large differences in scope, granularity and underlying formalism, ranging from term lists, over informal thesauri, e.g., medical subject headings (MeSH) [16], single-hierarchy classification systems such as ICD-10 [17] and formal ontologies such as SNOMED CT [15, 18].

Biomedical CVs have known drawbacks: some of them have a broad scope (covering all medicine), but lack the granularity required by particular clinical specialties or services. Others are restricted to a specific semantic category like diseases or drugs. Even those that provide good conceptual coverage of a domain, often lack sufficient lexical coverage, in particular for languages other than English. In fact, some studies have found out that semantic features recognized using CVs in clinical narratives are useless for some classification problems, probably because they are too broad [19]. Because of that, research projects often end up creating their own vocabulary [20–24], which is, e.g. documented by the large number of sources in repositories such as the UMLS Metathesaurus and BioPortal.

The predominance of the English language in biomedical publications is reflected by the fact that many CVs are restricted to English, whereas others have only partial translations to other languages. The Unified Medical Language System UMLS [25, 26] has been addressing these problems for several decades by linking common identifiers to 200 international clinical terminologies, thus extending the representation of medical terms in several languages. More recently, the creation of interface terminologies, separated but linked to reference terminologies has been emphasized [27]. Whereas reference terminologies are defined as representing, first, a domain in terms of formally or informally defined and language-independent representational units (concepts, descriptors, or classes), interface terminologies focus on the collection of clinical terms as used in practice, found in clinical narratives, with a focus on sub-language and user aspects.

### Contribution

Considering the availability of terminologies suited to cover a given clinical specialty in a certain natural language, a way to effectively apply NLP and ML technology to clinical narratives is to extend the scope of specialized language resources that are openly available for international and multilingual communities. Such resources are expected to accelerate the development and use of NLP and ML technology in the clinical domain, and to reduce the complexity of NLP tasks that deal with the severe problem of semantic (especially lexical) ambiguity, particularly in tasks like concept extraction [28], co-reference resolution [29] and domain-specific text classification.

This study scrutinizes the aforementioned problem from a Spanish language perspective. With its several dialects and varieties, Spanish is an official language in about 20 countries and is spoken by around half a billion people. Our source for Spanish is content from the literature database PubMed, for which we have proposed a method for automated harvesting medical term sets that are highly specific for a clinical specialty. Clinical specialties are subdivisions of the field of health care, such as represented by institutional divisions in health facilities, by fields of clinical research, and by undergraduate and post-graduate medical curricula. Instances of specialties are neurology, pathology, radiology, surgery and internal medicine, among many others, with sub-specialties like nephrology, diabetology, etc. There is no world-wide standard of clinical specialties, which explains high variations regarding subdivision of and overlaps between specialties.

The focus of this study is the development of a method for extracting lexical content from PubMed records, tagged by clinical specialties, and the creation of lists of terms that are specific for each specialty. The rationale is not to produce vocabularies that replace existing controlled terminologies but to support the production of specialized term sets, mainly for text classification purposes.

An additional contribution of this paper is to customize existing CVs to clinical specialties. This is the reason why we created SCOVACLIS (Spanish Core Vocabulary About Clinical Specialties).^1^

### Analysis of medical sublanguages in clinical narratives

Clinical sublanguage aspects have been addressed in many studies. Some of them identified differences in lexical and semantic patterns used within clinical specialties and types of clinical authors [30, 31], such as by applying clustering to a large set of clinical narratives using bag-of-words plus bag-of-UMLS features.

An early example is a method proposed by Bernhardt et al. [32], which identifies prominent clinical specialties linked to disease prevalence in the U.S. To this end, they connected mortality and morbidity information to medical literature. Epidemiology-related terms were extracted from national reports and standardized with MeSH terms.

In order to identify redundant information across specialties and clinical settings, Zhang et al. [33] automatically identified clinically relevant new information in inpatient and outpatient notes. Using semantic similarity techniques, they compared the language model extracted from a clinical note with the model extracted from previous notes from the same patient. Once they identified new and redundant information, they compared differences by specialty obtaining redundancy variations of 68.3% in inpatient notes compared to outpatient progress notes (60.7%). Pediatric notes exhibited the highest redundancy and radiology notes the lowest.

Finally, some studies [32, 34] applied supervised learning-based NLP to develop a medical subdomain classifier. They produced different classifiers and tested 105 combinations of data representations of the medical notes. Their main conclusion was that a “deep learning architecture with distributed word representation yielded better performance, yet the shallow learning algorithm”.

### Vocabulary extraction methods in healthcare

Harvesting vocabulary from biomedical literature has been subject to many studies in biomedical NLP research. In an early review, Krauthammer and Nenadic [35] distinguished three steps in a term identification process: term recognition, term classification, and term mapping. Meystre et al. [36] reviewed studies on clinical terminology extraction, most of which combined NLP techniques for term discovery with lexico-syntactic patterns for semantic relation discovery. The importance of terminologies to improve query expansion, information retrieval, ontology creation and data analysis was emphasized.

A method for extracting terms in a molecular biology context was described by Takeuchi and Collier [37]. The extracted terms were classified into ten semantic categories (e.g. protein, virus, cell type), using a support vector machine (SVM) model trained with a manually annotated MEDLINE abstract dataset. Regarding other medical sub-domains, some studies extract concepts that describe medical images [38] or medical curricula [39].

For languages other than English, Marciniak and Mykowiecka [40] harvested a list of single and multi-word terms used in Polish hospital discharge summaries. They observed that 70% of the obtained terms were not included in the Polish MeSH.

Finally, Sandoval et al. [41] created a biomedical corpus of validated terms from Spanish, Arabic and Japanese, by using several tools for optimal exploitation of the information contained in the corpus.

In this paper, we focus on the use of NLP techniques and tools for harvesting Spanish vocabulary from PubMed for a specific goal, *viz.* the construction of sets of terms that are maximally specific to clinical specialties. Such term sets can be useful for document classification, but also for adding new content to existing CVs. Our technique can be applied to any language as long as the related PubMed records include enough titles and links to abstracts in the original language. Subsequently, we will suggest a refinement for the term identification task, for which different statistical measures will be proposed in order to improve the selection of candidate terms. These measures are based on the importance of each term in each specialty and its importance in the overall corpus. Finally, this list of terms is used as an extra feature in a multi-label classifier.

## Methods

### Overview

**Fig. 1:**
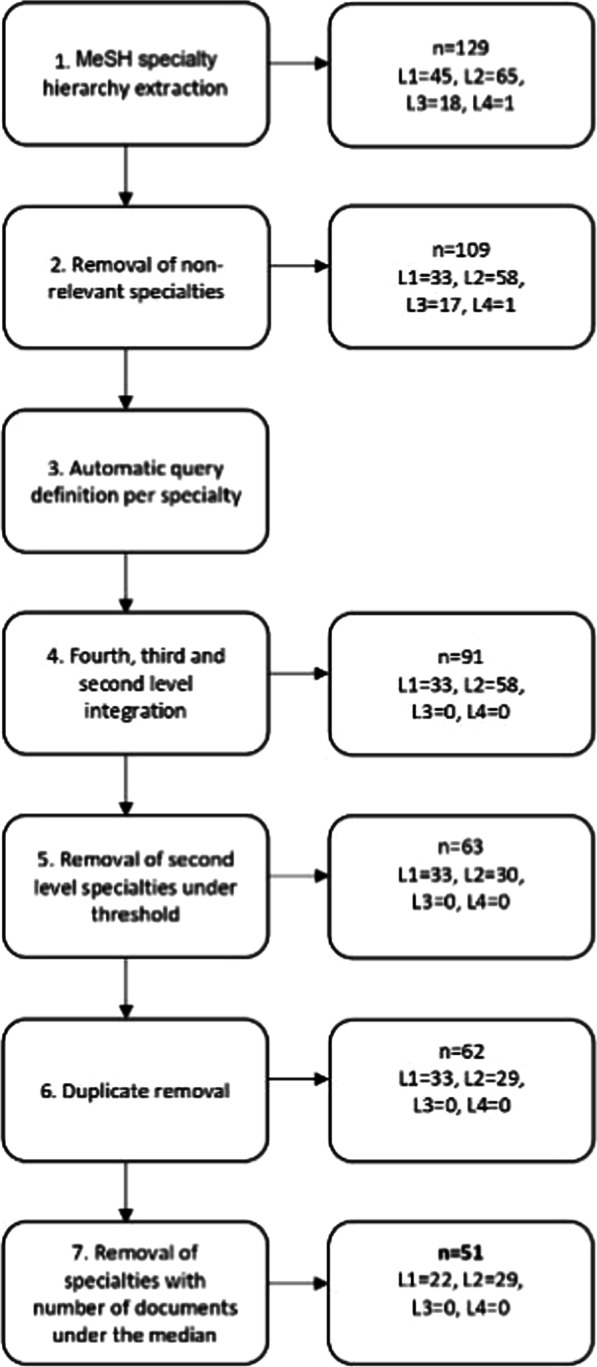
Overview of the extraction method

The method was designed to extract terms that are both *frequent in* and *specific for* a clinical specialty, thus resulting in a maximally characteristic term set for each clinical specialty. The balance between term frequency discriminative power then would result, e.g., for clinical oncology, in the selection of the important and frequent terms “*carcinoma*” and “*tumor*”, but also of less frequent, but highly specific terms like “*leucemia mielomonocítica*” or “*dermatofibrosarcoma*”. The main phases of this method are depicted in Fig. 1.

- The first phase of our method is the acquisition of domain corpora classified by clinical specialty (cf. "Specialty-specific corpus acquisition" section). Clinical texts might be the best source, but de-identified and therefore shareable clinical corpora, particularly for languages other than English do not exist, a well-known problem in biomedical NLP. This was the reason we decided to use Spanish content from PubMed, aware of the known terminology mismatch between scientific clinical language. For the selection of PubMed content by clinical specialty, several sources were combined. Aware that MeSH annotations would not suffice to indicate the clinical specialty to which a PubMed record belongs, we also included authors’ affiliation as a source of specialty-related information as a group of terms directly related to the specialty (e.g. “skin disease” for dermatology).
- The second phase (cf. "Term extraction" section), term extraction, yields word n-grams from PubMed titles and abstracts. Not all n-grams are good term candidates, therefore this phase contains an important automatic cleansing step.
- The last phase, term consolidation (cf. "Term consolidation strategy" section), identifies the importance of each previously identified word n-gram for the chosen sub-language and, according to this analysis, applies a filtering algorithm that produces a final term set for each clinical specialty. The filtering algorithm detects and removes those n-grams that are common to all or almost all clinical specialties and whose relevance to those clinical specialties is similar. These “stop n-grams” are useless for differentiating between specialties.

### Specialty-specific corpus acquisition

**Fig. 2:**
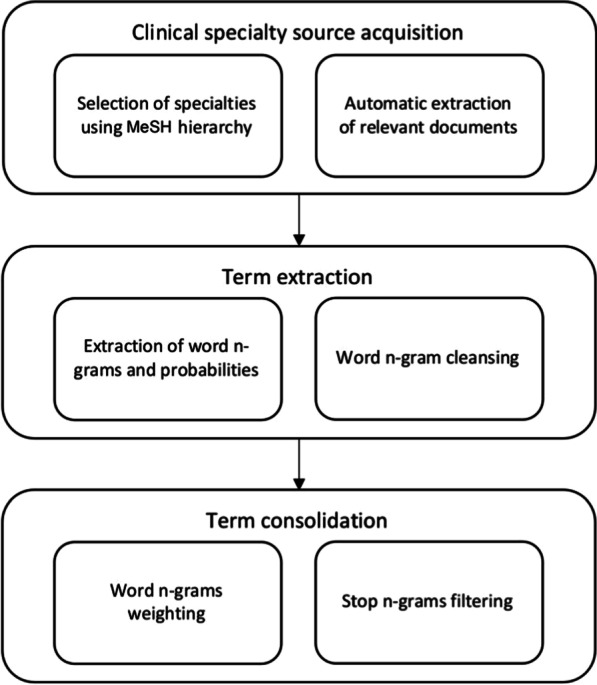
Clinical specialty selection process. Including the variations in the number of specialties (left) when applied to the Spanish case (right)

Figure 2 illustrates this phase in detail. The number of clinical specialties was 129 in the beginning (*n*). *L* refers to the number of specialties per hierarchical level. Out of 129 specialties, 45 belonged to the first level (*L1*) and 65 belonged to the second level (*L2*) of the hierarchy.

For defining the set of clinical specialties, the method starts extracting the branch of the MeSH thesaurus under “Disciplines and occupations” > “Health Occupations” > “Medicine” (Step 1).

Most subcategories obtained under this branch are clinical and paraclinical specialties, but others on education, research, epidemiology, or public health, were considered out of scope because they do not produce routine, non-research, textual content in health care scenarios (Step 2). Examples are “aerospace medicine”, and immunochemistry (a subspecialty of allergology and immunology). Another category considered as non-specialties, such as “clinical medicine”—an umbrella term for all clinical specialties, but in fact, it is orthogonal within the taxonomy, including “precision medicine” and “evidence-based-medicine” - none of them being clinical specialties that would produce a distinct kind of textual data and therefore be relevant for specific vocabulary generation. So, this branch was excluded as well.

Subcategory selection was the only step done manually, given the resources available, the inherent complexity of subdividing clinical specialties, the idiosyncratic nature of the division of the medical realm and the apparent lack of principled modeling of the specialty subtree in MeSH.

Out of this specialty selection, we generated the queries for retrieving specialty-specific PubMed content (Step 3), under the assumption that a text belongs to a clinical specialty *S* and is, therefore, relevant for term extraction if it fulfills one of the following conditions:

- The affiliation of the first author corresponds to an institution or department associated with *S*.
- The article was written for a publication in the area of *S*.
- The article was categorized using keywords relevant to *S*.
- The article was indexed with MeSH terms relevant to *S*.

The rationale behind these criteria is that the articles that contain specialty-relevant terms are rarely ever indexed with a MeSH term from the clinical specialty subtree. E.g., “RF-New Recombinant Vaccine for the Prevention of Herpes Zoster” is an article relevant for dermatology, but it is not annotated with the MeSH term “Dermatology”. This is also obvious when counting only 20,000 articles indexed with the MeSH term “cardiology”, opposed to 830,000 ones with the word “heart” in title or abstract. Following our goal, we exclusively analyzed Spanish content; a mandatory condition is that the PubMed record contains at least a Spanish title.

**Fig. 3:**
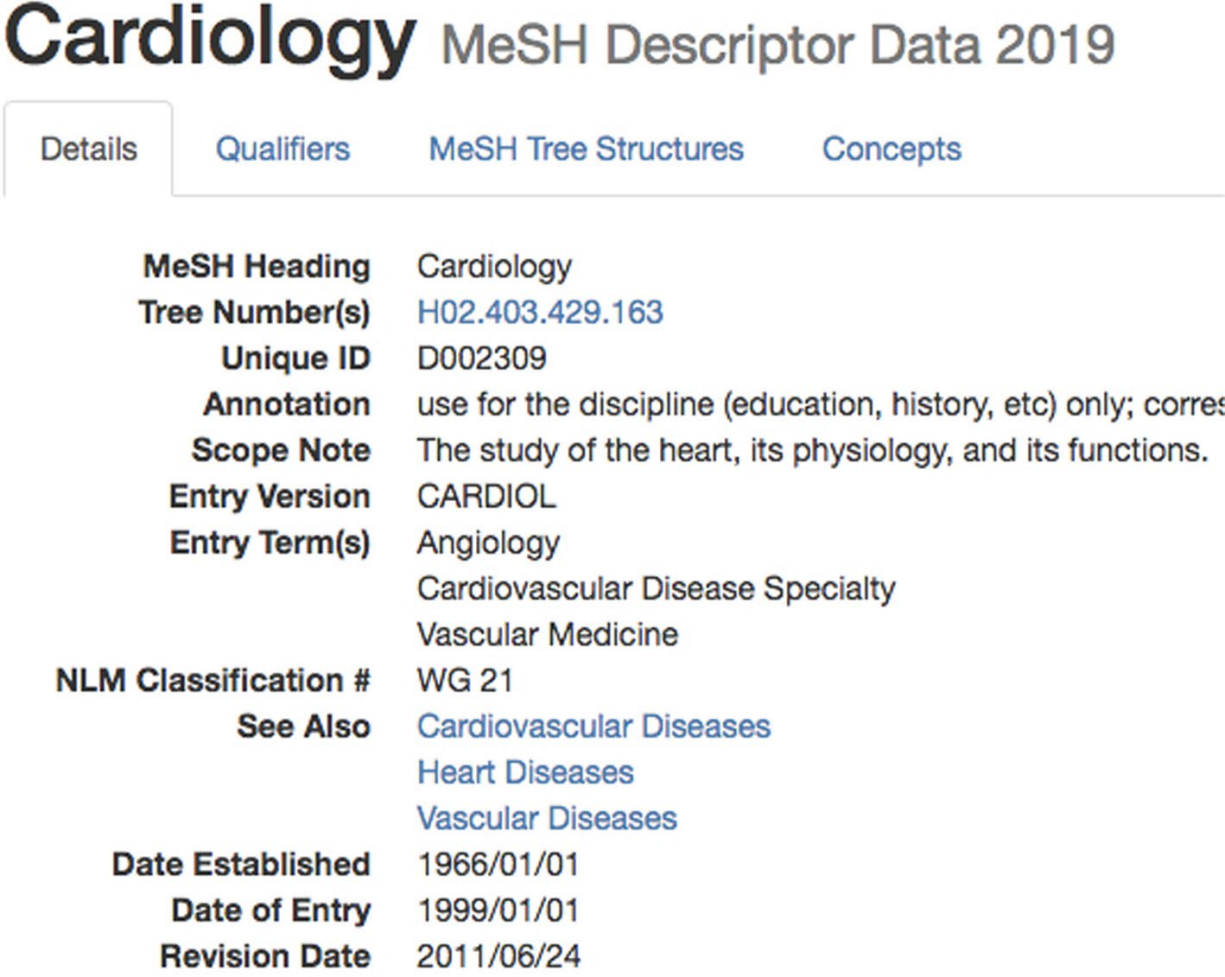
Example of MeSH term information

Our query generators (Step 3) performed PubMed queries using Biopython [42] for extracting specialty-specific corpora. The terms used to query for clinical specialties were extracted from the MeSH hierarchy. First, the MeSH term denoting the specialty itself, which is then expanded by the terms available via “See Also” links in MeSH, suggesting other specialty-relevant MeSH terms, e.g., Fig. 3 shows the specialty “Cardiology”, where three semantically close MeSH terms are suggested under “See also”.

In order to expand our query further, we added for each of these terms (MeSH and “See also”) Spanish MeSH translations retrieved from the UMLS Metathesaurus, marked as MSHSPA. E.g., the MeSH term “General Practice” is expanded by *Medicina General* for the MeSH ID D058006.

Despite the possibility to obtain more related terms like synonyms and term variants for describing a specialty, we decided to restrict ourselves to a limited set of cue terms that optimally delineate the scope of a clinical specialty. This strategy should maintain a high precision regarding the goal of our study, i.e. harvesting specific terms. It also allows easy reproduction of the query generation by using the UMLS Metathesaurus only. An example of the automatically generated query that selects dermatology content is the following:

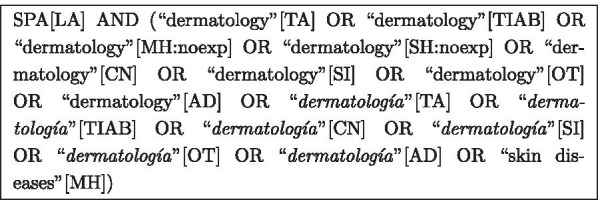



The following PubMed query demonstrates our interest in retrieving Spanish content from dermatology by selecting the following fields and values in the PubMed record:

- Has Spanish(SPA) as document language (LA).
- Contains “dermatology” or “dermatología” in at least one of the following fields: translated journal title (TA), title or abstract translated to English (TIAB), corporate author (CN), secondary source (SI), as keyword set by the author (OT), in some affiliation (AD), in MeSH Terms (MH:noexp) or MeSH Subheadings (SH:noexp).
- Finally, we include the terms “See Also”, in this case “Skin Diseases”, in the query to search for them in the MeSH terms [MH].^2^

Since many sub-specialties share the terminology preferences of the specialty from which they are derived, our method integrates terminology extracted from more fine-grained specialties to entries belonging to a higher level in the specialty hierarchy (Step 4).

Harvesting important terms that are highly specific for a specialty requires a considerable amount of text. The number of required texts may vary according to the diversity of terms for each specialty; however, a minimum amount of texts is desirable. For this reason, the process removes all second-level specialties with less than 1000 Spanish titles (Step 5) and then maintains only one specialty when it has duplicates, which means that it belongs to more than one MeSH subtree (Step 6). This happened with“Gynecology”, belonged to both the MeSH subtree “Reproductive Medicine” and “Specialties, Surgical”. The final step 7 eliminates specialties in the first level that have not yet achieved a significant amount of content.

From the final set of titles and abstracts per specialty, those with “case reports” as publication type were retained to be used as evaluation benchmark (i.e. test data set). The reason was that case reports seem to use a language that is closer to clinical narratives. Case reports typically document interesting observations on individual patients, e.g., new syndromes, particular and unusual evolution of diseases (often orphan diseases), complications of common treatments as well as beneficial or adverse effects of common or unusual therapies [43].

### Term extraction

Once we obtained a collection of Spanish titles and abstracts per specialty, all texts were then submitted to a process that extracts possibly relevant terms.

Term candidates were word n-grams with *n* between 1 and 3. E.g., from “*Manejo práctico de inmunosupresores en dermatología*”, the unigrams extracted are: *Manejo*, *práctico*, *de*, *inmunosupresores*, *en*, *dermatología*. The bigrams extracted are: *Manejo práctico*, *practico de*, *de inmunosupresores,* *inmunosupresores en*, *en dermatología*. The trigrams are: *Manejo práctico de*, *practico de inmunosupresores*, *de inmunosupresores en*, *inmunosupresores en dermatología*. We decided not to include n-grams for $n>3$ due computational cost and expected low frequency of longer n-grams.

The first step was to split each text into sentences, the established sentence boundary being “.”, “;”, “?”. “!”. As expected, many n-grams were not terms in a strict sense, such as those beginning or ending with articles and prepositions (e.g. “de”, “en”, “manejo práctico de”, “inmunosupresores en”. Term cleansing removed those n-grams that match one of the following rules, which were formulated after the inspection of an n-gram sample:

- Unigrams included in a Spanish stopword list, punctuation marks and digits.
- Bigrams that include at least one stopword or punctuation marks.
- Trigrams that include at least two stopwords or punctuation marks or whose last position is a stop word.
- N-grams that include a country, region or nationality name.
- N-grams containing dates or years.

Finally, all n-grams were normalised to singular nouns, e.g. from “*hematomas*” to “*hematoma*”. During this phase, we also extracted the acronyms and their expansions organized by clinical specialty. The new acronym resource is composed of 619 classified acronyms, and the method that we employed to built it is explained in detail in [44].

### Term consolidation strategy

In this last step, we applied a term weighting strategy that characterizes the n-grams that belong to a clinical specialty and then filters n-grams below a threshold, we refer to them as stop n-grams. The term weighting and filtering strategies consider the following restrictions:

1. *TF = 1 challenge* The volume of titles and abstracts from Spanish articles, furthermore split by clinical specialty, was just too small for applying the TF-IDF measure [45]. In most cases, the term frequency was just one, which is known as the TF = 1 challenge [46].
2. *Multi-class texts* A text may belong to more than one specialty, e.g., endocrinology and nephrology, which increases the overlapping of sets of specialty-related terms.
3. *Multipurpose vocabulary* Most proposals for weighting terms (or characteristics) have a well-defined application, such as text classification [47] or query expansion [48, 49]. Since we want to generate a raw set of terms that can serve several purposes, the characterization must take into account different needs. Some applications may require to have only the most discriminating terms for a specialty, others may require its most representative terms, even if they are also frequent in other specialties.

### Term weighting

Taking into account the aforementioned restrictions, the weighting strategy considered the following three measures:

- *Term global measure (TGM)* a corpus measure, which quantifies the concentration of a term along with all specialties.
- *Local precision measure (LPM)* a specialty-level measure, which represents the specificity of a term for a specialty. Accordingly, high LPM values characterize terms that never or rarely occur in texts belonging to other specialties.
- *Local relevance measure (LRM)* it represents the capacity of a term to describe the specialty. Terms with high values are those with high frequency in the specialty, compared to other terms in the specialty, and compared to the frequency in other specialties.

The three measures contribute to addressing the “TF = 1 challenge” restriction because the relevance of a term is not computed by text, but by the specialty. In addition, the ability to distinguish the importance of the same term for different specialties and the corpus as a whole deals with the “multi-class texts” restriction.

Similarly, the proposed local measures include two types of terms: (1) infrequent ones with a high predictive power (e.g., “*celoteioma*” for oncology), and (2) terms that are not only very frequent in text of the specialty, but also in other texts (e.g., “*cáncer*”or “*quimioterapia*” for oncology). Although unspecific they are relevant for the specialty. The differences between these measures contribute to deal with the “multipurpose vocabulary” restriction (see "Term consolidation strategy" section). The notation used to define these measures is explained in Table 1.

**Table 1:** Method notation

Notation	
*t*	The term under scrutiny
*N*	The total number of texts in the corpus
$N_{e_i}$	The number of texts belonging to the specialty *i*
$N^{t}$	The number of texts that contain the term *t*
$N^{t}_{e_i}$	The number of texts belonging to a specialty *i* that contain the term *t*
$E^t$	The number of specialties that contain the term *t*
$fe^t_i$	The number of occurrences in texts of the specialty *i* of the term *t*
$F^t$	The number of occurrences of the term *t* in all texts (Spanish abstracts and titles harvested from PubMed records) of all specialties

1 $$\begin{aligned} TGM(t) = 1 + \sum _{i=1}^{k} \frac{\frac{fe^t_i}{F^t} \times log \frac{fe^t_i}{F^t}}{log(N)} \end{aligned}$$

The global measure defined in Eq. 1 is a derivative of the entropy measure that evaluates the level of disorder or unpredictability, given a set of classes and a set of features [50]. A value of 1 would correspond to terms that exclusively occur in text from the specialty, whereas 0 would correspond to terms that are spread equally across texts belonging to all specialties. Whenever a term occurs in more than one specialty, a higher value is assigned to those terms whose difference in distribution among specialties is high (e.g. $fe^{t}_{1}=20$, $fe^{t}_{2}=2$, $fe^{t}_{3}=1$, $fe^{t}_{4}=3$, $fe^{t}_{5}=1$) and lower values when the difference is low (e.g. $fe^{t}_{1}=3$, $fe^{t}_{2}=5$, $fe^{t}_{3}=2$, $fe^{t}_{4}=1$, $fe^{t}_{5}=0$).

2 $$\begin{aligned} LPM(t,i) = \frac{N^{t}_{e_i}}{N^t} + \frac{N^{t}_{e_i}}{N_{e_i}} \end{aligned}$$

The first part of Eq. 2 measures the distribution of the term among the specialties and the second part evaluates the local importance using the negative texts within the specialty, i.e., the texts that do not contain the term.

If the term occurs in the texts belonging to a single specialty only and appears in all texts of this specialty the value of this measure will be greater. The global part range from 0 to 1 and the local from 0 to 1/($N_{e_i} - N^{t}_{e_i}$). Higher values are assigned to those terms that are highly specific for the specialty but can be rare.

3 $$\begin{aligned} \text {Given } L = P_{90}\left( \{ \forall F^t ; t \in e_i \} \right) \\ LRM(t,i)= {\left\{ \begin{array}{ll} \frac{N^{t}_{e_i}}{N^t}, & \text {if}\ F^t \ge L \\ 0, & \text {otherwise} \end{array}\right. } \end{aligned}$$

Typically, the measures used for assessing the importance of a term in a class penalize frequent terms that belong to different document classes because of their inability to accurately discriminate between classes. However, potentially important terms may be revealed considering their frequency in conjunction with their probability in the specialty and their probability in the corpus. The last measure defined in Eq. 3 evaluates this importance. *L* corresponds to the value of the 90% percentile of the global frequency value of terms that belong to the specialty *i*.

#### Filtering of stop n-grams

The last step filters the stop n-grams using the *LRM*(*t*, *i*) measure and the algorithm presented in 1.

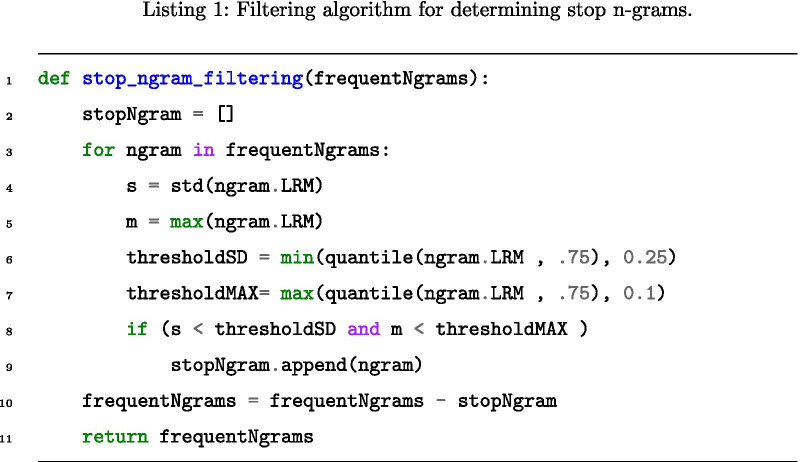



The hypothesis behind the algorithm is that an n-gram is irrelevant if its importance in all specialties where it appears is similar and if there is no specialty where its importance is considerably high. The input of the algorithm is the collection of frequent n-grams, i.e., n-grams appearing in more than 47 specialties. Using this collection, the algorithm detects the stop-n-gram candidates by evaluating the standard deviation of its LRM and the maximum value of its LRM across all specialties where the N-gram appears. When an n-gram has a standard deviation lower than a *thresholdSD* and a maximum value lower than a *thresholdMAX*, it is considered a stop n-gram. We selected the threshold of 47 specialties as it allows us to identify n-grams that are in more than 90% of the available specialties. This, combined with the analysis of importance metrics, allowed us to identify empty n-gram that should not be covered in the resource.

## Results

The proposed method was applied to create a set of Spanish terms called SCOVACLIS. For the validation of our initial hypothesis, this resource was used to address a text classification problem.

The following sections present the analysis of the method and the results of a text classifier using SCOVACLIS. The text classification experiments were made using the complete SCOVACLIS resource and a reduced version containing only the terms that are also found in SNOMED CT.

### Extraction of indicative terms for SCOVACLIS

#### Source acquisition

**Fig. 4:**
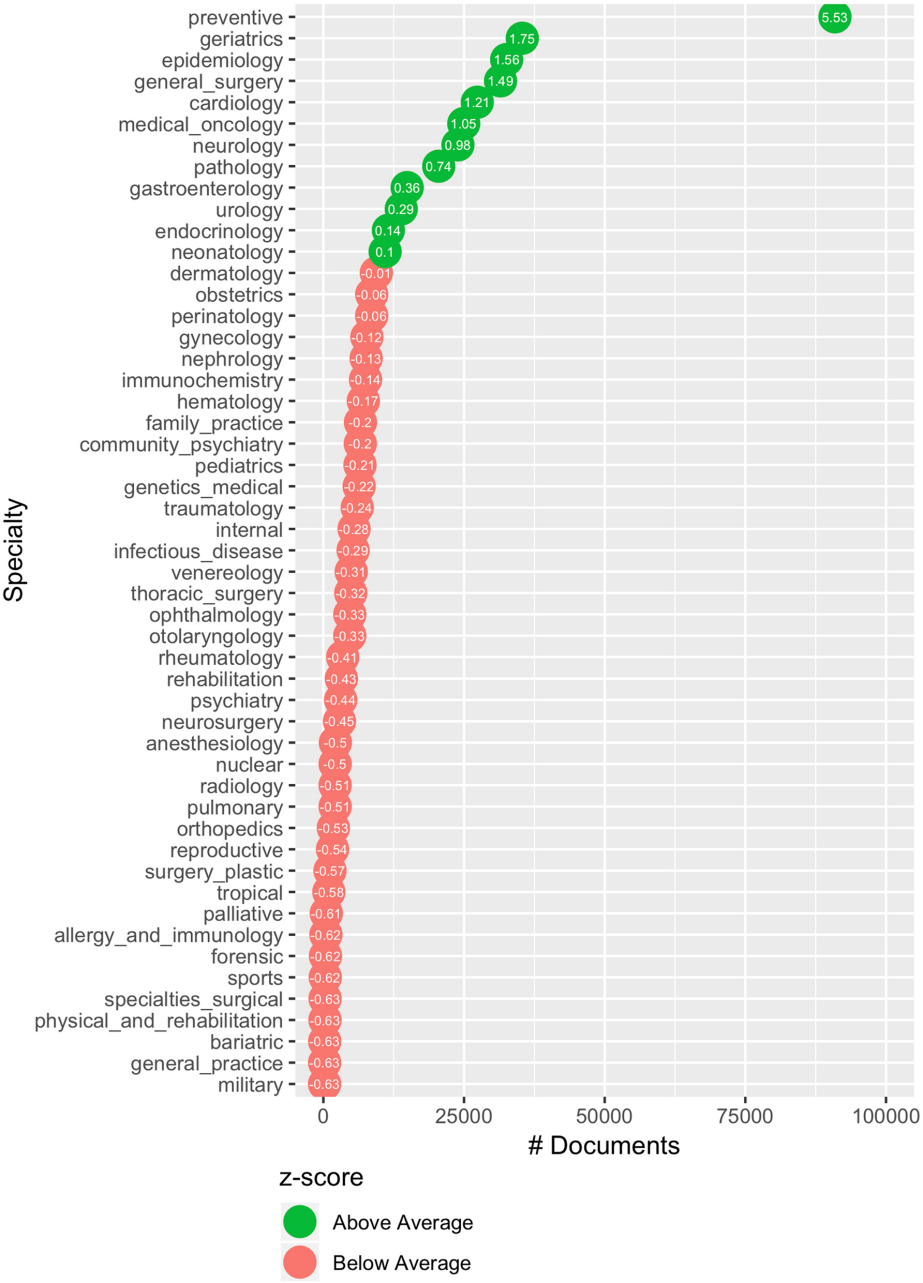
Terms per specialty and number of Spanish PubMed titles and abstracts. The value inside the point indicates how many standard deviations a specialty is away from the mean (a.k.a. z-score). The average number of tokens in the titles is 13.3, in the abstracts is 249.72

The first phase consisted of the acquisition of a corpus, consisting of Spanish titles and abstracts belonging to 51 clinical specialties. Figure 4 describes the number of available titles and abstracts for each specialty.

#### Term consolidation

The consolidation step yielded 635,699 n-grams. The most frequent ones across the specialties are presented in Table 2. Considering their provenance, these terms give an impression of the dominating research areas in the Spanish-speaking community, under the assumption that the rate of publishing in Spanish does not differ between communities.

**Table 2:** Most frequent n-grams and clinical specialties in which they appears. Riesgo: risk, población: population, atención primaria: primary care

N-gram	Total frequency	Specialties in which the n-gram mainly occurs
Cáncer	21,545	Medical oncology, preventive medicine, geriatrics, pathology, general surgery
Riesgo	15,796	Preventive medicine, epidemiology, cardiology, geriatrics, general surgery
Salud	14,608	Preventive medicine, epidemiology, community psychiatry, geriatrics, family practice
Renal	13,884	Urology, nephrology, preventive medicine, general surgery, geriatrics
Evaluación	10,655	Preventive medicine, geriatrics, epidemiology, general surgery, cardiology
Población	9592	Preventive medicine, epidemiology, geriatrics, cardiology, endocrinology
Tumor	8598	Medical oncology, pathology, preventive medicine, geriatrics, general surgery
Virus	7997	Preventive medicine, epidemiology, venereology, immunochemistry, medical oncology
Carcinoma	7973	Medical oncology, pathology, geriatrics, preventive medicine, general surgery
Atención primaria	7526	Family practice, preventive medicine, geriatrics, epidemiology, cardiology
Factor de riesgo	6747	Preventive medicine, epidemiology, cardiology, geriatrics, endocrinology
Mortalidad	6572	Preventive medicine, epidemiology, geriatrics, neonatology, cardiology
Arterial	6425	Cardiology, preventive medicine, geriatrics, epidemiology, nephrology
Programa	6216	Preventive medicine, community psychiatry, epidemiology, geriatrics, family practice
Trasplante	6103	General surgery, preventive medicine, urology, nephrology, thoracic surgery
Pronóstico	6040	Preventive medicine, geriatrics, medical oncology, cardiology, pathology
Insuficiencia	5995	Cardiology, preventive medicine, nephrology, urology, geriatrics

**Fig. 5:**
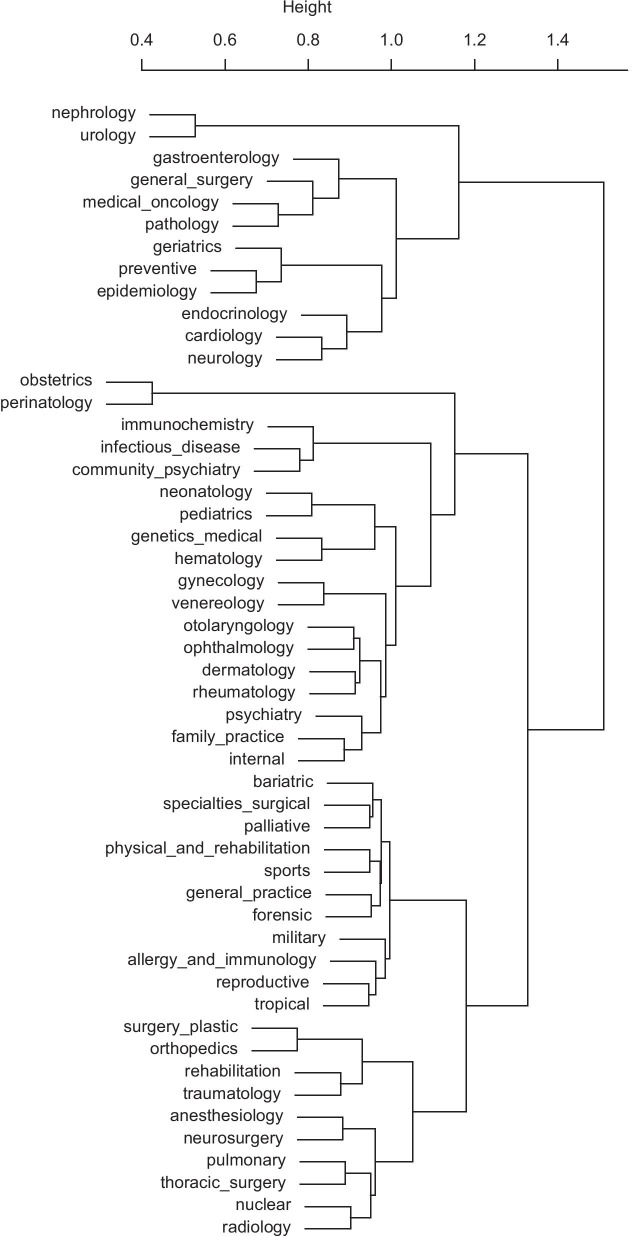
Similarity between specialties according to their n-grams

As expected, some specialties overlap. Figure 5 visualizes the similarity between clinical specialties. That the three most similar pairs of specialties are obstetrics—perinatology, nephrology—urology and preventive medicine—epidemiology is evident from the closeness and partial overlapping of these disciplines.

### Medical text classification enriched using SCOVACLIS

In order to test the hypothesis that NLP tasks benefit from more specific terminologies, such as SCOVACLIS, we assessed how much a text classification task can be improved by using the new vocabulary.

We propose a multi-label classification. In this classification, the goal was to classify case reports, extracted from PubMed, by one or more clinical specialties to which they belong. To create the gold standard, a PubMed query with publication type “Case report” and language “Spanish” retrieved 54,881 PubMed records with Spanish titles, of which 714 also had a Spanish abstract. Our classification uses both titles and abstracts, counting on average 11.28 and 163.23 tokens, respectively.

We used 75% of the case reports for training (41,159 texts) and the rest for blind testing (13,720 texts). Our available repository contains more information about the distribution of labels for each partition (train and test) created for this evaluation.^3^ For the primary evaluation standard metrics from the ML and NLP community were used: micro-averaged precision (P), recall (R), and balanced F1-score (F1).

In addition, taking into account SNOMED CT as a reference terminology in Spanish, we have performed two experiments, (1) using the complete SCOVACLIS and, (2) filtering SCOVACLIS with terms covered by SNOMED CT.

#### Multi-label classification

Because a PubMed record can be related to one or more clinical specialties, the experiment involved multiple labels per case report (title and abstract). Labels are binary variables that indicate class (i.e., clinical specialty) membership. This scenario implies greater difficulty due to the computational cost of model generation and querying, as well as the presence of unbalanced labels. In ML, the first step towards training a classifier is text pre-processing, where feature extraction and vectorization take place.

The entire implementation was done using Python on a single Tesla-V100 32 GB GPU with 192 GB of RAM. We performed experiments with different classifiers that would allow multi-label classification such as:

- *Random Forest* [51] In our experiment, the number of trees taken into account is 100, as a function of measuring quality we use entropy.
- *K-nearest Neighbors* [52] The number of neighbors used in our experiment is 5, the weight function used in the prediction is uniform (all points in each neighborhood are weighted equally), and the other default parameters.
- *Decision Tree* [53] Similar to the parameters used in Random Forest, here we also use entropy as a function to measure the quality of a split and the other default parameters.
- *Multilayer Perceptron (MLP)* [54] a multilayer perceptron network consisting of three layers: (1) one input layer, (2) one hidden layer, and (3) one output layer. The number of nodes in the hidden layer is 100, use reLU activation, 0.001 in learning rate and use early stopping for controlling overfitting.

We tested different parameters to adjust the classifiers to the problem (for detail you can refer to our repository.^4^) We also performed tests with different word representation vectors such as TF-IDF, one-hot encoding and bag-of-words. The best results were obtained using TF-IDF with the following parameters: lowercase = True, stopwords = Spanish stop words and n-gram range = (1,3).

To validate the usefulness of SCOVACLIS, we added features as extra information to each text, using a vector with size 51 according to the number of clinical specialties. The value of each feature is a score calculated following Eq. 4.

4 $$\begin{aligned} SCOVACLIS_s (d) = \sum _{i=1}^{n} TGM(t) , \forall t \in s, \end{aligned}$$

where:

- *d* is the document (in our case a title or an abstract)
- *n* is the number of n-grams
- *s* is the specialty

The results obtained by the different combinations of classifiers and word representation are shown in Table 3.

**Table 3:** Multi-label classification. Annotated data results with SCOVACLIS Score ($SCOVACLIS_s$) and removing stop-ngrams ($SCOVACLIS_s$—stop ngrams)

Classifier	Word representation	P (%)	R (%)	F1 (%)
Random forest	TF-IDF	71.7	25.1	38.4
Decision tree	TF-IDF	47.9	38.1	42.4
KNeighbors	TF-IDF	63.3	39.0	48.2
MLP	TF-IDF	75.1	53.3	59.3
Random forest	TF-IDF + $SCOVACLIS_s$	70.0	17.5	28.7
Decision tree	TF-IDF + $SCOVACLIS_s$	46.2	43.5	44.8
KNeighbors	TF-IDF + $SCOVACLIS_s$	69.3	42.6	52.7
MLP	TF-IDF + $SCOVACLIS_s$	74.7	57.4	64.9
Random forest	$SCOVACLIS_s$	76.0	32.6	45.6
Decision tree	$SCOVACLIS_s$	42.6	43.1	42.8
KNeighbors	$SCOVACLIS_s$	69.4	42.1	52.4
MLP	$SCOVACLIS_s$	75.8	43.5	55.3
Random forest	TF-IDF + $SCOVACLIS_s$—stop ngrams	70.3	18.9	30.6
Decision tree	TF-IDF + $SCOVACLIS_s$—stop ngrams	46.4	43.9	45.1
KNeighbors	TF-IDF + $SCOVACLIS_s$—stop ngrams	68.9	42.7	52.7
MLP	TF-IDF + $SCOVACLIS_s$—stop ngrams	**77.5**	**57.7**	**65.2**
Random forest	$SCOVACLIS_s$—stop ngrams	76.8	32.6	45.8
Decision tree	$SCOVACLIS_s$—stop ngrams	43.1	43.1	43.1
KNeighbors	$SCOVACLIS_s$—stop ngrams	68.9	42.7	52.7
MLP	$SCOVACLIS_s$—stop ngrams	75.6	43.5	55.4

In the KNN, Decision Tree and MLP classifiers the use of the term set as a feature improved the baseline (TF-IDF). The 10% increase using the MLP method stands out, achieving 64.9% using TF-IDF with the created collection (TF-IDF + $SCOVACLIS_s$).

In the second scenario, in which we used only the term set ($SCOVACLIS_s$), we observed a small increase in almost all cases except the MLP classifier.

In the third scenario (TF-IDF + $SCOVACLIS_s$—stop ngrams) we removed stop-ngrams. We observed the same as in the first scenario (TF-IDF + $SCOVACLIS_s$), so we improved the base case in most cases. However, the difference with the first scenario was small, which led us to conclude that the stop-ngrams did not add too much noise to the classification. Here we achieved the highest precision and recall score, with an F1 measure of almost 65.2% using MLP (results in bold).

Finally, the scenario in which we removed stop-ngrams ($SCOVACLIS_s$—stop ngrams) obtained values similar to the second scenario ($SCOVACLIS_s$).

We concluded the study by observing that the term set improved the baseline in both cases, using it alone or including it as a set of features in the classifier (see more details in GitHub.^5^)

SCOVACLIS has been generated with the aim of improving vocabulary resources taking into account clinical specialties. Currently, no standard or reference terminology classifies terms per clinical specialty, so no direct comparison can be made. However, to analyze the relationship of our resource with curated terminology, we performed experiments using SNOMED CT.

The goal is to quantify the level of overlapping between SCOVACLIS and SNOMED CT, and to analyze whether the non-overlapped terms in SCOVACLIS were useful to perform a task that requires discrimination per clinical specialty. To this end we downloaded the international SNOMED CT release in Spanish (2020-04-30 release) and performed the same pre-processing as for our term set ("Term extraction" section).

For this experiment, SCOVACLIS was reduced for each clinical specialty, taking into account only those terms that were included in SNOMED CT. The average number of terms covered by SNOMED CT in all specialties was 28.16%. E.g., dermatology has been reduced from 46,000 terms to around 12,000 and cardiology from 110,000 to around 20,000 terms. With this resource we repeat the classification experiments and applied them to the test data set.

We analyzed the characteristics of the terms that were not covered by SNOMED CT in order to recognize possible noise terms in our term set (for more details you can refer to our results.^6^) We could demonstrate that many SCOVACLIS terms with high $E^t$ values (cf. "Term weighting" section) were found in SNOMED CT. A high $E^t$ means that the term was found in several specialties. 90% of the terms found between 38 and 52 specialties were included in SNOMED CT, in contrast to only 15% of the terms found between 1 and 13 specialties. This corroborates that the Spanish SNOMED CT gives preference to terms of wide-spread use but lacks coverage of very specific terms only used in one specialty. This seems interesting, because SCOVACLIS could be used for adding more content wherever an NLP task requires these specific and complementary terms. The manual analysis of the terms yields that some of the missing terms are related terms to formal ones (e.g. “filled breast” instead of “breast implant” in “Surgery plastic”), and others are more specialized than the ones contained in SNOMED CT (e.g. “thoracoabdominal wall” in “Thoracic Specialty” and “kayakista” in “Sports Medicine”). Lastly, we found that specialties with a broad lexical nature, such as preventive medicine and general practice, apparently include terms of minor relevance. However, they are useful for improving classification performance, as illustrated in the classification experiment.

**Table 4:** Multi-label classification. Annotated data results with SCOVACLIS Score ($SCOVACLIS_s$) and filtered terms with SNOMED CT

		Original			SNOMED CT filter		
Classifier	Word representation	P (%)	R (%)	F1 (%)	P (%)	R (%)	F1 (%)
Random forest	TF-IDF + $SCOVACLIS_s$	70.0	17.5	28.7	69.1	14.0	22.6
Decision tree	TF-IDF + $SCOVACLIS_s$	46.2	43.5	44.8	37.3	34.0	35.8
KNeighbors	TF-IDF + $SCOVACLIS_s$	69.3	42.6	52.7	62.8	31.1	41.6
MLP	TF-IDF + $SCOVACLIS_s$	74.7	57.4	64.9	70.1	56.1	60.5
Random forest	$SCOVACLIS_s$	76.0	32.6	45.6	65.4	14.0	23.1
Decision tree	$SCOVACLIS_s$	42.6	43.1	42.8	31.5	26.8	28.9
KNeighbors	$SCOVACLIS_s$	69.4	42.1	52.4	57.9	29.0	38.7
MLP	$SCOVACLIS_s$	75.8	43.5	55.3	73.4	24.3	36.6

Finally, Table 4 shows the comparison between the classification results with the original term set (*original* columns) and with the reduced term set restricted to terms occurring in SNOMED CT (*SNOMED CT filter* columns). The latter, reduced term set does not improve the original classification. Particularly, when taking into account the F1 measure, we obtained a reduction between 4 and 18 points. This means that the terms included in SCOVACLIS improve the recognition of the specialties, even though they are not canonical or curated terms.

#### Label distribution analysis

Considering that the datasets used for training and testing were not balanced regarding the specialty, this section analyzes the classification error versus the distribution of the class by dividing the F1 results into ranges: 0 to 25%, 25% to 50%, 50% to 75% and 75% and above. 18 specialties with an average of 356 texts for training, obtained an F1 value of less than 25%. nine specialties (including, e.g. geriatrics and psychiatry), obtained an F1 between 25 and 50%, with an average of 2049 texts for training and 688 for testing. Specialties such as pathology and obstetrics and 13 others had an F1 between 50 and 75%, with an average of 2635 training texts. Finally, the specialties that reported the best results (i.e., over 75%) were nine, with oncology and cardiology among them, having on average, 5915 titles/abstracts to train the system. In the first range (i.e., 0–25%), there were six specialties with F1 = 0; they contained less than 30 training titles/abstracts and less than seven titles/abstracts for testing, among them were forensic medicine, general practice and allergy, and immunology.

This analysis allows the conclusion that, as expected, the more texts the system has for learning, the better it classifies. Our multi-label approach makes the task more problematic as demonstrated by the following misclassifications examples:

Example #1.

Text: Infiltración pleural en la recaída de un mieloma múltiple.

Translation: Pleural infiltration in multiple myeloma relapse.

True specialties: cardiology, hematology, medical oncology, pathology and preventive medicine.

Predicted specialties: cardiology, hematology, medical oncology and pathology.

Example #2.

Text: Osteoartropatía hipertrófica en adenocarcinoma de pulmón.

Translation: Hypertrophic osteoarthropathy in lung adenocarcinoma.

True specialties: medical oncology, pulmonary medicine and rheumatology.

Predicted specialties: medical oncology.

Example #3.

Text: Derivación gástrica en Y de Roux como procedimiento de urgencia para resolver la fuga en un SADI-S.

Translation: Roux-en-Y gastric bypass as an emergency procedure for resolving SADI-S leak.

True specialties: bariatric medicine and general surgery.

Predicted specialties: bariatric medicine, general surgery and gastroenterology.

We highlight the difficulty that our classifier has to detect specialties considered transversal (i.e., that do not have a very specific vocabulary) such as pathology, internal medicine or general surgery (cf. detailed results.^7^)

## Discussion

Term identification is crucial for many tasks of automated biomedical text processing of biomedical [7–14], with CVs as fundamental resources [15]. We have proposed and validated a method not only to harvest terminology from texts but to classify texts by clinical specialties.

The application of this method to a *Spanish Core Vocabulary About Clinical Specialties* (SCOVACLIS) is the first accomplishment of this research. The results obtained allow us to recommend this method for obtaining specialized clinical term sets in any language sufficiently represented in PubMed titles and abstracts. Our approach proved useful for recognizing frequent, infrequent, and equally relevant terms that are characteristic of given clinical specialties. The completeness of the term sets obtained by our method is directly related to the volume and richness of the texts obtained for each specialty. It was not surprising that broad, overarching specialties such as general practice or preventive medicine contained less specific terms. and, consequently, underperformed in text classification. Our goal is not to replace existing controlled terminologies, but to support the production of specialized term sets tailored to tasks that require terms with high predictive value, as they occur in clinical or research texts, regardless of naming conventions used for the building of CVs. In contradistinction to these intensively curated resources, the emphasis of our method is on producing term sets in a fully automatic manner, which may include non-standard terms, e.g. with custom abbreviations or frequent spelling variants. This, however, does not exclude at all the potential of our method to provide useful input to terminology developers who maintain and extend clinical terminologies, particularly interface terminologies [27], which focus on the language used in research or clinical documentation. For the latter purpose, real-world clinical would be preferable as input, for which a good data protection strategy is indispensable. Unfortunately, nearly all de-identified clinical document collections that are publicly accessible, e.g. MIMIC III [55], are in English so that no sufficient amount of publicly available Spanish clinical text could be obtained. Other methods need a manually labelled corpus [37] or use nomenclature rules to detect terms that do not exist in large lexicons such as UMLS [56]

A resource named SCOVACLIS is the second accomplishment of our research. It allows us to enrich the set of resources available for NLP in Spanish. Creation and validation of SCOVACLIS support the hypothesis that a term set classified by clinical specialties might reduce the level of ambiguity when compared to a specialty-independent, broad-scope vocabulary. Disambiguation, particularly of short forms, is a known bottleneck in clinical text processing.

Regarding the validation of SCOVACLIS, its use for improving the features in a multi-class classification approach using a Multilayer Perceptron classifier achieved an increase in 6 percentage points in the F1-measure compared to the baseline. This shows its usefulness to improve contextual knowledge about medical texts and thus better solve NLP problems such as named entity recognition and classification. Also, SCOVACLIS demonstrated an increased text classification performance, compared to the use of curated terms such as the ones included in SNOMED CT, currently the largest source of Spanish clinical terminology. Sotelsek and Villena [57] obtain similar results with their MIDAS system that assigns ICD-9-CM codes to radiology reports. In their case they use a manually developed lexicon of words, multiwords and abbreviations to help their system.

Implementing this method and producing the language resource was not straightforward; were several challenges had to be addressed. Also for the creation of SCOVACLIS, the inevitable restriction to the terminology used in titles and abstracts of research papers is a known limitation, because of its difference from the jargon found in clinical documents, which are known to be hastily written and marred with typos and cryptic short forms. To what extent SCOVACLIS is a useful resource for handling clinical documents still has to be investigated. For future work, we plan to expand our term set and use comprehensive reports.

We also had to find criteria to decide which clinical specialties to use; our solution, mainly based on the MeSH occupation hierarchy enriched by other features extracted from PubMed metadata, is more complex than it would have been with clinical texts, whose provenance (clinical department in the document header) would have been trivial to ascertain. In contradistinction, an easily accessible source of biomedical texts is the literature database MEDLINE, with PubMed as a free search engine. From more than 26 million records approx. 2.2 million are about non-English publications, including about 330,000 Spanish entries for which a Spanish title and sometimes a Spanish abstract is available. By using the publication type “case reports” for evaluation, we have extracted a publication genre that is supposed to be closer to clinical language than other, purely scientific papers.

Thanks to the existence of freely available query interfaces to PubMed and MeSH, the process of obtaining relevant texts for each specialty was executed. The fact that the universe of publications linked to a medical specialty in MEDLINE is much larger than the set of articles indexed by an occupation-specific MeSH term, led us to enrich the search method by incorporating new elements that allow us to increase the recall of the query.

In contrast to related works [36], the extraction of texts as input for term extraction is done automatically, including classification by specialty. Our manual effort was limited to the crafting of the search strategy using MeSH terms and text words in the authors’ affiliations. Therefore, our method can be applied in any language that has available scientific publications in PubMed. [37, 39]. Likewise, it can be used to create term sets for different domains, depending on the initial descriptors [40, 41].

Even though we used the Spanish titles and abstracts to create the term set, we found a limitation because only 4592 PubMed records were linked to an automatically accessible abstract in Spanish. Thus, most of the texts under scrutiny consisted of titles only. The use of Spanish full texts could be a solution, with about 64,000 Spanish full text sources being freely accessible from PubMed. This, however, would require considerable manual effort.

For future work, we plan to explore word embeddings and train some to use them in traditional ML [58] or neural network approaches. In addition, there are available pre-trained models for the biomedical domain, such as BioBERT [59], we could consider. Although BioBERT is in English, an ideal scenario would be the generation of a new model for Spanish trained by a large biomedical corpus. It is also important to evaluate the value of using specific terminologies in NLP tasks involving specialties with a broad lexical nature, such as preventive medicine and general practice.

The research hypothesis chosen and tested in this paper was that SCOVACLIS improves classification of medical texts. That SCOVACLIS might also be useful for other NLP tasks is only being discussed, but this could be a subject of future research, given its usefulness for text classification. That our task is fully automated does not exclude human judgements in the evaluation process. MEDLINE is annotated by human experts; our gold standard is largely grounded on these annotations, which we can consider of good quality.

## Conclusions

The following objectives were pursued with this study: first, to elaborate a method for extracting non-English content from PubMed records; second, to tag these extracts by clinical specialty; and third, to generate characteristic term lists for each clinical specialty.

The method for the automatic extraction of medical terms involves the following steps:

1. Selection of clinical specialties to be evaluated.
2. Generation of a PubMed extract (titles and abstracts) labeled by clinical specialties.
3. Extraction of word n-grams.
4. Normalization of word n-grams.
5. Generation of metrics that support the selections of the relevant terms in each clinical specialty.
6. Identification of stop n-grams.

The resource obtained by applying this method to Spanish titles and abstracts, named SCOVACLIS, was evaluated in a multi-label classification task. The results have shown that our resource improved the baseline (without SCOVACLIS). We obtained an F-measure of 65.2% using an MLP network, 77.5% in precision and 57.7% in recall using TF-IDF representation, and SCOVACLIS without stop n-grams as features.

Finally, the creation and validation of SCOVACLIS support the hypothesis that specific term sets classified by clinical specialty might reduce the level of ambiguity when compared to a specialty-independent and broad-scope vocabulary.

## Data Availability

The datasets generated and/or analysed during the current study are available in the GitHub repository, https://github.com/plubeda/scovaclis

## Footnotes

1 *https://github.com/plubeda/scovaclis*.

2 https://www.ncbi.nlm.nih.gov/books/NBK3827/table/pubmedhelp.Tn/.

3 https://github.com/plubeda/scovaclis/blob/master/Distribution-of-labels.md.

4 https://github.com/plubeda/scovaclis/blob/master/Detailed-classifiers.md.

5 https://github.com/plubeda/scovaclis/blob/master/Detailed-results.md.

6 https://github.com/plubeda/scovaclis/blob/master/SNOMED-CT-analysis.md.

7 https://github.com/plubeda/scovaclis/blob/master/Binary-confusion-matrix.md.
